# Virtual reality-guided therapy on a stroke unit: a feasibility study

**DOI:** 10.1186/s42466-024-00357-w

**Published:** 2024-12-02

**Authors:** Jordi Kühne Escolà, Rumeysa Demirdas, Martin Schulze, Woon Hyung Chae, Lennart Steffen Milles, Doreen Pommeranz, Marvin Darkwah Oppong, Christoph Kleinschnitz, Martin Köhrmann, Benedikt Frank

**Affiliations:** 1 Department of Neurology and Center for Translational Neuro- and Behavioral Sciences (C-TNBS), University Hospital Essen, Hufelandstr. 55, 45147 Essen, Germany; 2 Department of Physical Therapy, University Hospital Essen, Essen, Germany; 3 Department of Neurosurgery and Spine Surgery and Center for Translational Neuro- and Behavioral Sciences (C-TNBS), University Hospital Essen, Essen, Germany

**Keywords:** Virtual reality, Stroke, Functional recovery, Stroke unit, Physical therapy, Rehabilitation

## Abstract

**Background:**

VR (Virtual Reality) has emerged as a recent treatment approach in neurorehabilitation. The feasibility of VR-guided therapy in the acute phase after stroke has not been assessed.

**Methods:**

This was a cohort study of consecutive patients with suspected stroke who were admitted to the Essen University Hospital Stroke Unit between March 2022 and May 2022. All patients who had an indication for physical or occupational therapy due to upper extremity sensorimotor, cognitive or perceptual deficits were included and considered for VR-guided treatment. We excluded patients with predominant deficits in lower extremity function, since these could not be targeted with our VR system. A multidimensional approach was used to assess the feasibility of VR-guided therapy, which included characterization of eligible patients, resource utilization as well as treatment acceptance. For this purpose, we analyzed baseline and clinical characteristics, causes for withholding the treatment as well as qualitative and quantitative treatment metrics in patients who received VR-guided therapy.

**Results:**

Out of 326 patients admitted with suspected stroke, *n* = 172 were included in our final analysis. Of these, *n* = 37 (21.5%) received VR-guided therapy. The most common cause for withholding treatment were neuropsychological limitations (22.9%), followed by physical impairment, comorbidity and level of consciousness alterations (all 17.8%). Patients who received VR-guided therapy tended to have better functional status and less severe neurological deficits. VR-guided sessions had a median duration of 20 min (IQR 17–29) with additional 13 min (IQR 9–17) of preparation time. In the majority of patients who received VR-guided therapy, motivation was rated equal or higher as compared with conventional treatment (76%) and therapists considered VR-guided therapy well feasible (65%).

**Conclusions:**

Despite important treatment barriers, VR may provide additional opportunities to enhance functional recovery in the acute phase after stroke for selected patients. Our findings could aid in planning further randomized controlled trials which are required to refine approaches and assess the effectiveness of VR-guided therapy in the acute setting.

## Introduction

Virtual reality (VR) enables users to experience environments that appear and feel similar to the real world but have been created through technology [1]. Interaction and immersion in these virtual environments facilitate task-specific exercise with multisensory engagement that can be recorded, measured, and tailored to individual needs. Thus, among many other areas of application, VR provides opportunities to enhance rehabilitation [2]. As a result, VR-guided strategies have emerged as a novel treatment approach to improve functional recovery and reduce impairment in neurological disorders [3, 4].

VR-guided therapies address various aspects of neurorehabilitation, such as patient motivation, engagement and adherence [5]. Furthermore, authors have suggested positive effects on neural plasticity [6, 7]. VR can also be used to facilitate and enhance established therapeutic approaches such as mirror therapy [8, 9]. Another area of application is combination of VR with telemedicine, which allows patients to engage in rehabilitation programs remotely and thus might also enable better resource utilization [10, 11]. Such approaches could help to close gaps between acute Stroke Unit treatment and limited availability of post-acute rehabilitation services, however, differences in health care systems must be considered.

Although VR-guided strategies in neurorehabilitation seem auspicious, many of the underlying mechanisms are not completely understood and their clinical impact is still uncertain. While numerous approaches have been tested, some of the firmest evidence for application of VR-guided therapies in neurorehabilitation exists for recovery of upper extremity function after stroke. A recent overview of systematic reviews on this topic suggests that VR with or without conventional therapy is superior to conventional therapy on the Fugl Meyer Assessment scale for upper extremity (FMA-UE) [12]. However, it remains largely elusive when, how and in which patients VR-guided strategies might be most beneficial.

The vast majority of clinical evidence on VR-guided neurorehabilitation has emerged from post-acute studies. Yet, in neurological disorders and stroke in particular, current treatment paradigms emphasize early initiation of rehabilitative care [13]. While VR might offer additional opportunities to enhance and diversify early functional recovery in neurological disorders, its application in an acute neurological setting has not been studied so far. Thus, our aim was to determine whether VR-guided therapy is feasible on a Stroke Unit and to highlight treatment barriers as well as potential opportunities by characterizing eligible patients, treatment metrics and reasons for withholding VR-guided therapy.

## Methods

### Study Design

We analyzed consecutive patients who were admitted to the Essen University Hospital Stroke Unit between March 2022 and May 2022. As part of clinical routine, patients with a suspected diagnosis of stroke are screened by physical and occupational therapists upon admission to our Stroke Unit. All patients over 18 years of age who had a potential target for VR-guided treatment defined as upper extremity sensorimotor, cognitive or perceptual deficits with an indication for physical or occupational therapy were included. Patients who had predominant lower extremity impairment and in whom gait training was the main therapeutic objective were excluded from our analysis, since our VR-guided approach was not applicable for these deficits. This study also excluded patients who had no clinical neurological deficit upon initial screening and thus had no indication for physical or occupational therapy. Initiation of VR-guided therapy was considered in all patients who were included in addition to conventional treatment. We pursued a multidimensional approach to assess the feasibility of VR-guided therapy on our Stroke Unit. This included characterizing eligible patients as well as treatment barriers, resource utilization and acceptance by patients and caregivers. In case VR-guided treatment was not administered due to medical conditions, severe functional impairment or any other reason at the discretion of the treating therapist (e.g. patient preferences or therapeutic goals), a detailed description was required. These patients were treated with conventional treatment alone. VR-guided treatment was provided via CUREO® software (CUREosity GmbH, Düsseldorf, Germany). CUREosity GmbH was not involved in this study and did not provide any funding. VR-guided treatment sessions were administered at the bedside under one-to-one supervision by a physical or occupational therapist (no group or remote treatment). Three sets of hardware components were available. Each of these consisted of a head-mounted Oculus Quest device (Meta Platforms Technologies, Irvine, CA, USA), two controllers and a tablet serving as an interface for therapists to control treatment sessions, which allowed them to simultaneously provide hands-on assistance when needed. VR-guided treatment was provided via six different therapy modules (*Stimulate and activate attention*, *Motor training of the upper extremities*, *Cognitive and sensory training*, *Fine motor skills of fingers and hands*, *Neuro-regulation and -relaxation*, *Practice of everyday skills*). Modules could be selected deliberately by the treating therapists, based on the patient’s predominant clinical deficit and therapeutic goals. All therapy modules contained a large variety of clinically validated exercises. These consisted of playful tasks in a virtual environment with visual as well as auditory feedback and different degrees of difficulty. As an example, one exercise for upper extremity motor function included catching meteors in a space-like environment at varying degrees of speed and range of motion. All patients in the VR-group underwent at least one VR-guided treatment session. However, there was no upper limit for the total amount of sessions administered during the hospital stay. After VR-guided treatment sessions, therapists completed a standardized questionnaire to record time metrics and the patient’s perceived effort (on a numeric rating scale from 0 to 10, graded as very low [0–2], moderate [3–5], vigorous [6] and very intense [7–10]). Therapists were also asked to rate patient motivation (as compared to conventional treatment), feasibility, and autonomy in performing the treatment (all on a five-point visual analog scale ranging from no agreement to strong agreement). Data from the standardized questionnaires was then linked to the patient’s medical records including diagnosis, comorbidities and stroke symptoms at admission and discharge as recorded by National Institutes of Health Stroke Scale (NIHSS). Functional status before admission, at admission and at discharge was estimated according to the modified Rankin Scale (mRS). The study was approved by the local ethics committee (approval number 22-10534-BO).

### Statistical Analyses

We report descriptive statistics of baseline and clinical characteristics for all patients in whom treatment with VR was considered. In addition we describe reasons why VR-guided therapy was not performed as well as a metrics of VR-guided treatment session. Categorical data are reported as counts and percentages, while continuous data is described as median (interquartile range, IQR). Fisher’s exact or Mann–Whitney-U-test were used as appropriate for comparing baseline characteristics of patients who received VR-guided therapy and those who did not despite initial consideration. All statistical tests were two sided, and *p* values of < 0.05 were considered statistically significant. No adjustments for multiple testing were made, since this was an exploratory analysis and findings should be interpreted as preliminary. Statistical analyses were carried out with SPSS, version 29 (IBM Corp., Armonk, NY, USA).

## Results

**Fig. 1:**
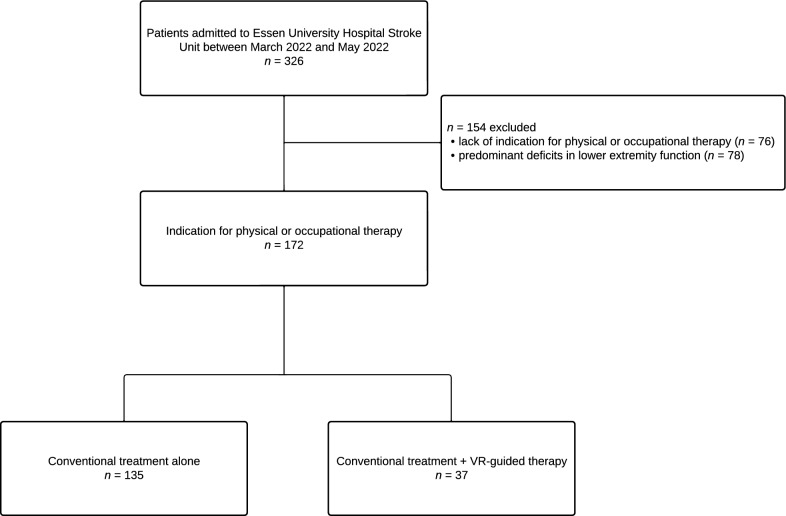
Flow chart of study population. *VR* Virtual reality

Between March 2022 and May 2022, 326 patients were admitted to our Stroke Unit due to suspected stroke (Fig. 1). After excluding patients without an indication for physical or occupational therapy and those with predominant lower extremity deficits, *n* = 172 were included in our final analysis. Of these, *n* = 37 (21.5%) received VR-guided therapy.

### Barriers of VR-guided therapy

**Fig. 2:**
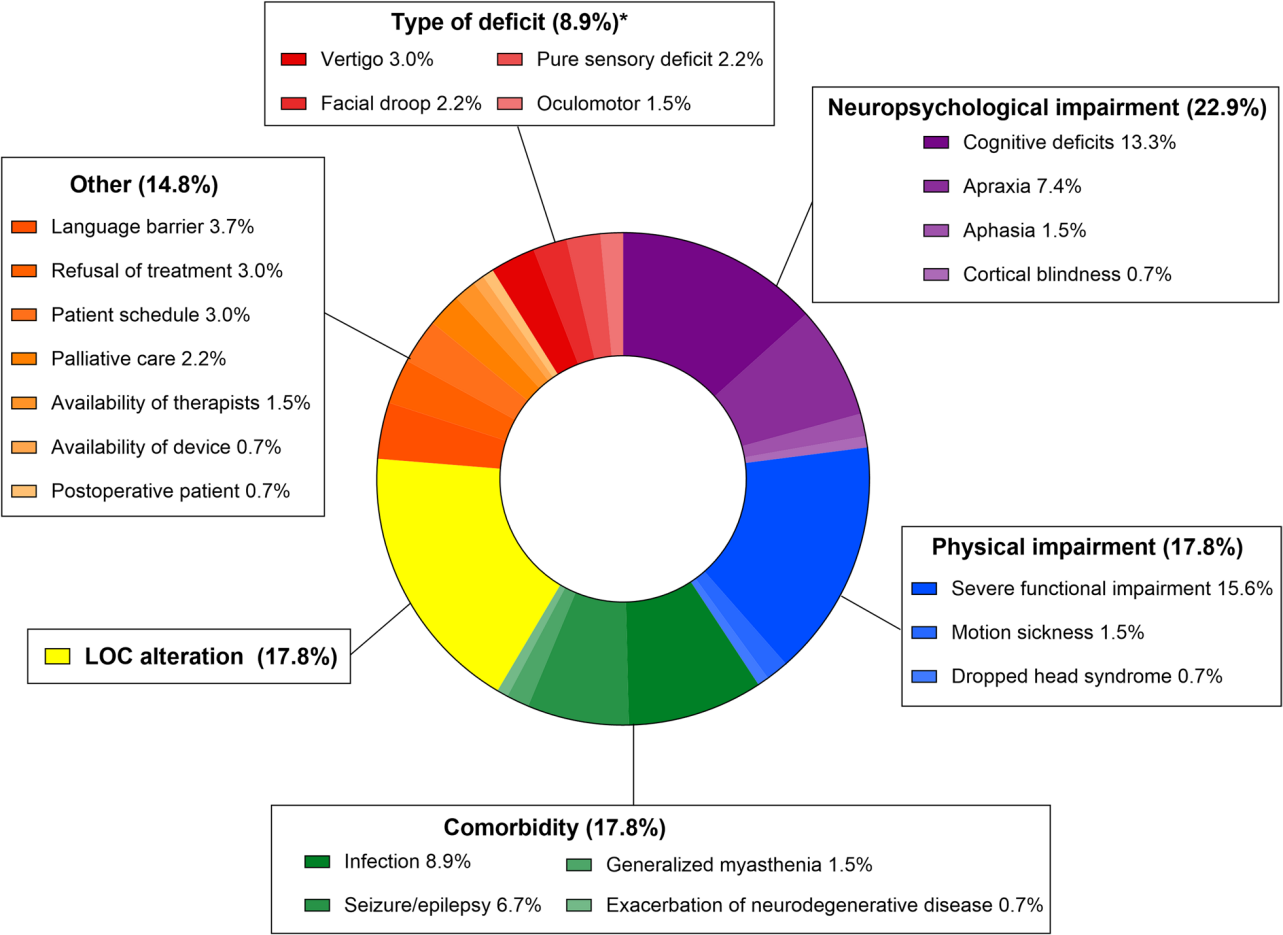
Reasons for withholding VR-guided therapy in *n* = 135 patients. *LOC* Level of consciousness. *Deficits in which therapists preferred conventional approaches over VR-guided therapy

Several reasons for withholding VR-guided treatment were recorded (Fig. 2). Most commonly, neuropsychological limitations impeded patients from participating in VR-guided treatment sessions (22.9%), followed by physical impairments, comorbidities and level of consciousness alterations (all 17.8%).

When comparing patients who were treated with VR-guided therapy and those who did not receive the treatment, no differences in baseline demographic data as well as underlying diagnoses were observed (Table 1). Patients who received VR-guided therapy tended to have better functional status prior to admission (median prehospital mRS 0.0, IQR 0.0–1.0 vs. 1.0, IQR 0.0–3.0; U = 1584, Z = − 2.6, *p* = 0.011), at admission (median mRS at admission 3.0, IQR 1.0–4.75 vs. 4.0, IQR 3.0–5.0; U = 1749, Z = − 1.8, *p* = 0.076) and at discharge (median mRS at discharge 2.0, IQR 1.0–3.75 vs. 3.0, IQR 1.0–4.0; U = 1712, Z = − 2.2, *p* = 0.027) as compared to those who underwent conventional treatment only. There was also a trend towards less severe neurological deficits upon admission as measured on the NIHSS in patients who were treated with VR (median NIHSS at admission 3.0, IQR 1.0–8.5 vs. 6.0, IQR 2.0–11.0; U = 1998, Z = − 1.9, *p* = 0.062).

**Table 1:** Baseline and in-hospital characteristics of patients screened for VR-therapy (*n* = 172)

	Conventional treatment alone (*n* = 135)	Conventional + VR-guided therapy (*n* = 37)	*p* value
Baseline characteristics			
Age (years), median (IQR)	72.0 (64.0–83.0)	68.0 (56.0–82.0)	0.187
Female sex, *n* (%)	72 (53.3)	17 (45.9)	0.462
Atrial fibrillation, *n* (%)	51 (37.8)	11 (29.7)	0.441
Diabetes, *n* (%)	37 (27.4)	7 (18.9)	0.396
Hypertension, *n* (%)	101 (74.8)	29 (78.4)	0.829
Prior stroke, *n* (%)	29 (21.5)	8 (21.6)	> 0.99
Prehospital grade of disability (mRS)^a^, median (IQR)	1.0 (0.0–3.0)	0.0 (0.0–1.0)	0.011
Diagnosis, *n* (%)			
Ischemic stroke or TIA	97 (71.9)	27 (73.0)	> 0.99
Intracerebral hemorrhage	21 (15.6)	6 (16.2)	> 0.99
Stroke mimic	17 (12.6)	4 (10.8)	> 0.99
In-hospital characteristics, median (IQR)			
Grade of disability (mRS) at admission^b^	4.0 (3.0–5.0)	3.0 (1.0–4.75)	0.076
Stroke severity (NIHSS) at admission	6.0 (2.0–11.0)	3.0 (1.0–8.5)	0.062
Grade of disability (mRS) at discharge^c^	3.0 (1.0–4.0)	2.0 (1.0–3.75)	0.027
Stroke severity (NIHSS) at discharge^d^	4.0 (2.0–11.0)	3.0 (1.0–9.0)	0.199
Length of admission (days)	6.0 (4.0–14.0)	8.0 (4.0–19.0)	0.119

*mRS* Modified Rankin Scale, *NIHSS* National Institutes of Health Stroke Scale, *TIA* Transient ischemic attack, *VR* Virtual reality

Data available in ^a^*n* = 120/135 & 36/37; ^b^*n* = 120/135 & 36/37; ^c^*n* = 125/135 & 36/37; ^d^*n* = 134/135 & 35/37

### VR treatment characteristics

**Fig. 3:**
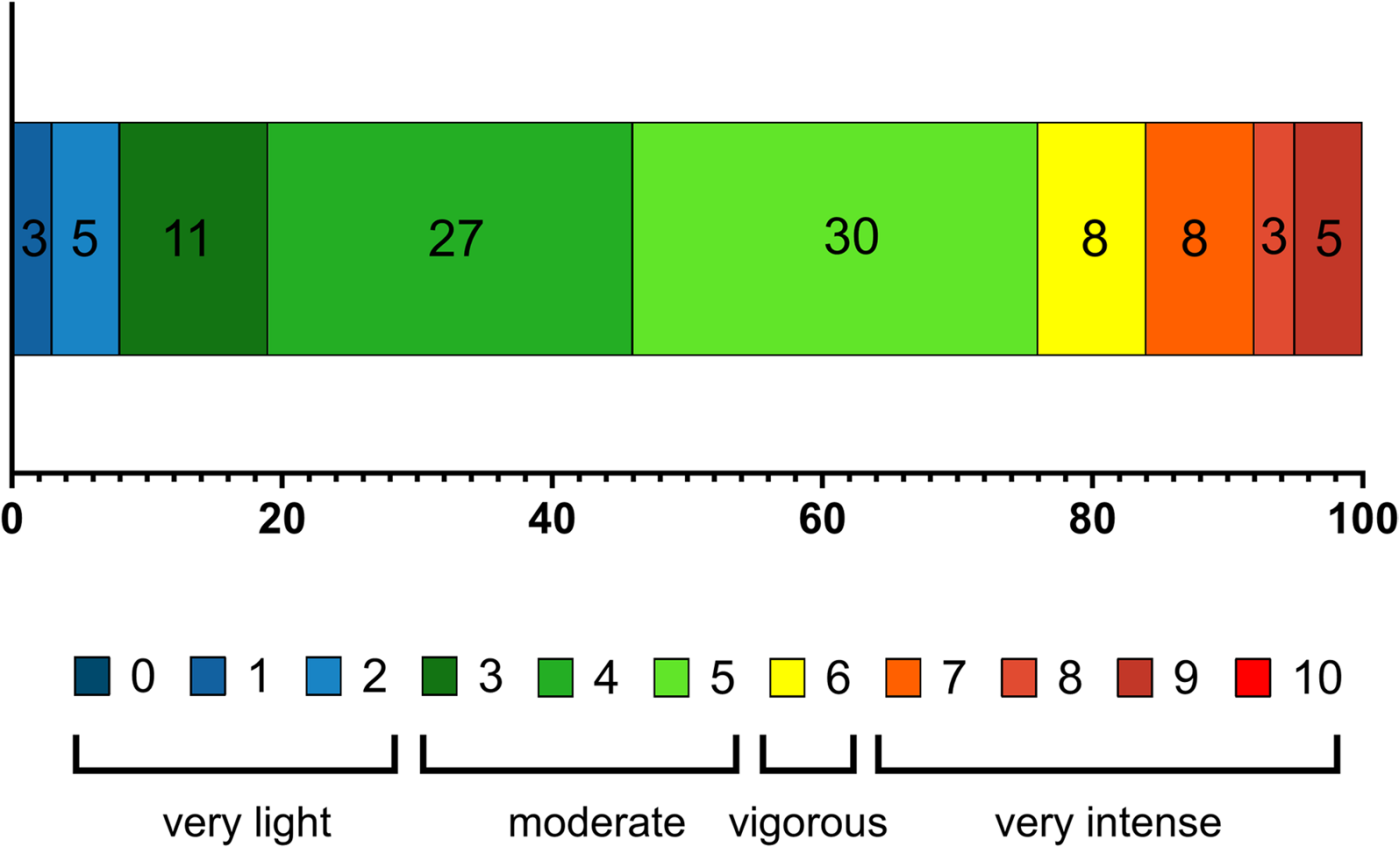
Distribution (%) of perceived effort during treatment sessions on a numeric rating scale from 0 (no effort) to 10 (maximum effort) in patients who received VR-guided therapy (*n* = 37)

**Fig. 4:**
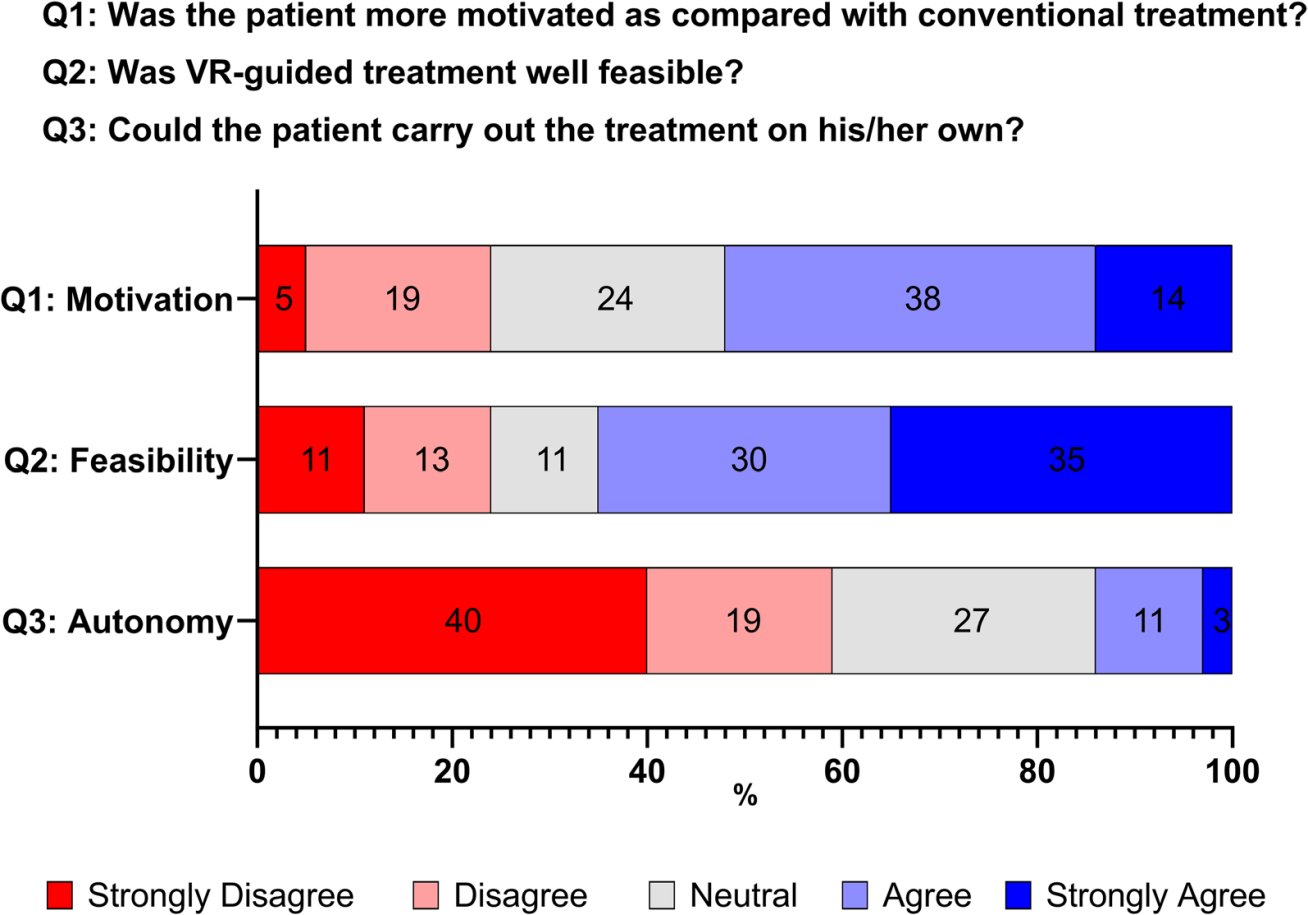
Distribution (%) of questionnaire responses (*n* = 37) regarding patient motivation, feasibility, and autonomy in performing VR-guided therapy, as rated on a five-point visual analog scale ranging from strong disagreement to strong agreement

A total of 37 patients successfully completed VR-guided treatment sessions with a median duration of 20 min (IQR 17–29) and additional 13 min (IQR 9–17) for device set-up. The median perceived effort was 5/10 (IQR 4.0–5.5) (Fig. 3). In the majority of cases who received VR-guided therapy, the treating therapists considered that the treatment was well feasible (65%) and rated patient motivation equally high or greater as compared with conventional treatment (76%) (Fig. 4). However, VR-guided sessions required close supervision and therapists considered that patients were not able to perform the treatment autonomously in most cases (59%).

## Discussion

We analyzed VR-guided therapy in an acute setting and our study revealed three major findings: First, more than half of patients who were admitted to our Stroke Unit for suspected stroke were potential candidates to receive treatment with VR. Second, there were substantial treatment barriers and only one in five patients actually received VR-guided therapy. Third, in the majority of patients who were treated with VR-guided therapy, motivation was rated equally high or greater as compared with conventional treatment and therapists considered the treatment well feasible.

Several barriers of VR-guided therapy were observed in our study, many of which resulted as direct consequences of an acute disease (mainly severe stroke or intracerebral hemorrhage). Treatment barriers included neuropsychological or physical deficits that impaired patients from actively participating in VR-guided treatment sessions as well as acute complications such as infections or seizures due to which patients were not considered stable enough to receive the treatment. As expected due to our patient selection, those who received VR-guided therapy correspondingly tended to have better functional status and less severe neurological deficits upon admission, although baseline risk factors or overall diagnoses did not differ between groups. VR-specific reasons for withholding or discontinuing treatment that have been described previously such as motion sickness due to VR exposure (“VR sickness” or “cyber sickness”) [14, 15] played a marginal role in our study (less than 2% of patients in whom VR-guided therapy was withheld). When further examining reasons for withholding VR-guided therapy, issues related to the integration of VR into clinical workflows seemed to be of minor importance first (device or personnel availability restrained treatment in less than 3%). However, we observed that up to a third of the time at the bedside had to be spent on preparation, instructions and gear mounting. Thus, integration of VR into clinical workflows may have significant effects on active treatment time and resource burden, especially when being applied on a large scale. While improvements might be achieved with growing experience, optimization of workflows and technical advancements, time requirements and resource utilization must therefore be considered as important factors when evaluating the use of VR-guided therapy in an acute setting.

While more than half of patients admitted to our Stroke Unit were initially considered as potential candidates for VR-guided treatment, patients with predominant lower extremity deficits had to be excluded from our analysis, since these impairments could not be targeted with our treatment approach. These observations reflect current standards of VR-guided treatment after stroke, which is largely focused on upper extremity function [16] and thus misses out an important subgroup of stroke patients. However, authors have introduced VR-guided approaches in patients with lower extremity deficits and studies on this subject are ongoing [17, 18]. Targeting lower extremity function would address an important limitation of many current VR treatment protocols in stroke. Hence, such new approaches may open up further therapeutic options for a large cohort of patients and offer potential to enhance early mobilization after stroke, which is crucial to maintain patient autonomy and prevent immobility-related complications.

Our findings also highlight further opportunities of VR-guided therapy in an acute setting. In the majority of patients who did receive VR-guided therapy on our Stroke Unit, the treatment was regarded well feasible with moderate to vigorous treatment intensity and a higher motivation as compared to conventional treatment was reported. Given that VR is considered safe, relatively inexpensive and can be tailored to individual patient needs [19], it could be a valuable tool to enhance functional recovery early after stroke, when effects of exercise might be most beneficial [20]. However, caution and further investigation are required, since the optimal timing and dosage of physical therapy after stroke remains uncertain and trials have found signals of potential harm in very early high dose mobilization [21]. Nonetheless, our findings suggest that a considerable subgroup of patients on a Stroke Unit could benefit from VR-guided approaches, especially when deficits are limited and predominantly affect upper extremity motor function.

Important limitations must be considered when interpreting our study. This was a single-center approach with overall low numbers, especially in the VR-guided therapy arm. Treatment allocation was non-randomized and VR-guided therapy could be withheld at the discretion of the treating therapists. Therefore, our study is prone to selection bias and we cannot exclude that factors such as time constraints or personal preferences have contributed to our observations. To facilitate the use of VR, our study allowed different therapy modules which may be heterogeneous in feasibility and this could also have affected findings. Subjective measures were used to characterize VR-guided treatment sessions, which can be highly variable among individuals and thus limit the generalizability of our results. For pragmatic reasons, assessment of motivation, feasibility and autonomy involved only one item each, which ensured simple and quick evaluation at the expense of comprehensiveness and reliability. Although we predefined a standardized questionnaire to report treatment, all data were collected during clinical routine and thus bear the risk of inaccuracy or incompleteness.

However, to our knowledge this is the first study to characterize the use of VR-guided therapy on a Stroke Unit. Despite important limitations, our analysis provides a general overview of treatment barriers and potential opportunities of VR-guided treatment in an acute setting. These findings may encourage clinicians to further implement and investigate VR-guided methods and also aid in planning further randomized controlled trials designed to assess treatment efficacy.

## Conclusions

Up to half of patients on a Stroke Unit could be potential candidates for VR-guided therapy. However, current approaches in the acute setting face substantial treatment barriers. Further studies should focus on refining patient selection, timing, and treatment protocols, in order to determine if and under which circumstances VR-guided therapy in an acute setting can be effective.

## Data Availability

The datasets used and analyzed for the current study are available from the corresponding author on reasonable request.

## Abbreviations

FMA-UE: Fugl Meyer Assessment scale for upper extremity
IQR: Interquartile range
mRS: Modified Rankin Scale
NIHSS: National Institute of Health Stroke Scale
VR: Virtual reality
